# Choosing your (Friedel) mates wisely: grouping data sets to improve anomalous signal

**DOI:** 10.1107/S205979831801570X

**Published:** 2019-01-31

**Authors:** Nicolas Foos, Michele Cianci, Max H. Nanao

**Affiliations:** a Structural Biology Group, European Synchrotron Radiation Facility, 71 Avenue des Martyrs, F-38000 Grenoble, France; b Department of Agricultural, Food and Environmental Sciences, Marche Polytechnic University, Via Brecce Bianche, 60131 Ancona, Italy

**Keywords:** single-wavelength anomalous diffraction, anomalous scattering, multi-crystal crystallography, serial crystallography, merging

## Abstract

Single-wavelength anomalous diffraction (SAD) phasing from multiple crystals can be especially challenging in samples with weak anomalous signals and/or strong non-isomorphism. Here, advantage is taken of the combinatorial diversity possible in such experiments to study the relationship between merging statistics and downstream metrics of phasing signals. It is furthermore shown that a genetic algorithm (GA) can be used to optimize the grouping of data sets to enhance weak anomalous signals based on these merging statistics.

## Abstract 2

Merging statistics are evaluated in multi-crystal single-wavelength anomalous diffraction phasing and are used to optimize the anomalous signal.

## 1. Introduction   

Although *de rigeur* before the advent of modern cryocooling methods, until recent years the assembly of a complete data set from multiple incomplete data sets had become less common. Recently, thanks in no small part to the development of methods for serial crystallography at free-electron lasers, there has been renewed interest in all aspects of multi-crystal crystallography. The use of multiple crystals allows much better data quality for a given X-ray dose because of the potential to use many hundreds or thousands of crystals. While this has previously been explored via microbeams and high-precision sample-manipulation devices (Cusack *et al.*, 1998 ▸), the scale and the nature of sample delivery has expanded tremendously. One of the key challenges associated with the use of multiple crystals is non-isomorphism. This is particularly relevant to anomalous phasing, especially in cases where the anomalous signal is very low. Based on previous work, it has been shown that better data can be obtained by merging subsets of data using, for example, correlation coefficients between sub-data sets or unit-cell parameter clustering (Santoni *et al.*, 2017 ▸; Foadi *et al.*, 2013 ▸; Liu *et al.*, 2013 ▸). For a handful of sub-data sets all combinations of sub-data sets can be evaluated, but this becomes unfeasible with even a relatively small number of data sets, since the number of combinations is 2*^n^* − 1 for *n* data sets. We have recently introduced an alternative approach in which a genetic algorithm (GA) is used to partition the pool of data sets into subgroups (Zander *et al.*, 2016 ▸; Foos *et al.*, 2018 ▸). The GA formulates the selection of isomorphous groups in evolutionary terms. The key concept is the encoding of the sub-data-set grouping in a data structure known as a chromosome. Here, a chromosome is an array of integers of length *n*, where *n* is the number of sub-data sets. The numeric value of each integer in the array encodes the merging group to which a sub-data set belongs. There is no limit to the number of groups that can be used, and the number can be chosen based on the number of non-isomorphous groups that are present in the data. In practice, it is common for only two groups to exist, but the default behaviour is to use three groups in case a third non-overlapping (and non-isomorphous) group exists (or, for example, two high-quality but non-isomorphous groups and a third ‘low-quality’ group of sub-data sets that do not merge with either of the two former groups). The first step of the GA is to randomly initialize a set of chromosome data structures. This population of chromosomes is then submitted to cycles of GA optimization, which consist of mutating positions in the array (changing the merging group), single- and double-crossover events between chromosomes, and evaluation of the ‘fitness’ of individual chromosomes. The ‘fitness’ is derived from the *R*_meas_ value, the 〈*I*/σ(*I*)〉 value, the CC_1/2_ value, the completeness, the multiplicity and the anomalous correlation coefficient. The relative weighting of these different components can be adjusted depending, for example, on the presence or absence of anomalous scatterers. Furthermore, default values have been determined which are generally effective. If anomalously scattering elements are present, this approach depends critically upon a connection between merging statistics and the ‘solvability’ of a data set. Considerable work has gone into studying the relationship between merging statistics and anomalous signal in single crystals, but less is known about the multi-crystal case. Although normally an impediment, we have taken advantage of the fact that sub-data sets can be assembled into single data sets in a large number of different ways. We use this fact to generate a large number of data sets with different merging statistics, and re-evaluate commonly used merging statistics in the context of multi-crystal data. In doing so, we show that the anomalous signal can be improved using the GA approach, that the anomalous correlation coefficient appears to be the best target for GA optimization and that this metric translates into improved downstream metrics such as anomalous difference-map peak heights, the ability to determine substructures and phasing success.

## 2. Methods   

### 2.1. Sample preparation   

Thermolysin crystals from *Bacillus thermoproteolyticus* and urease crystals from *Sporosarcina pasteurii* were prepared as described previously in Zander *et al.* (2016). Briefly, thermolysin was crystallized in 35% ammonium sulfate and was then cryoprotected with 2 *M* trimethylamine *N*-oxide. Urease was crystallized in 1.6–2.0 *M* ammonium sulfate in 50 m*M* sodium citrate buffer pH 6.3 and was then cryoprotected with 20% ethylene glycol, 2.4 *M* ammonium sulfate in 50 m*M* sodium citrate buffer pH 6.3. Cerulean crystals were grown using a microseeding method. A macrocrystal was obtained using the protocol described by Lelimousin *et al.* (2009 ▸). Macrocrystals were ground in 100 µl seeding buffer (0.1 *M* HEPES pH 6.75, 22% PEG 8000). The seeds were diluted to 1/100 in seeding buffer. The protein (15 mg ml^−1^) was digested with trypsin (0.5 mg ml^−1^) for 1 h [the ratio of trypsin to protein was 1:10(*v*:*v*)]. The seeds were mixed with digested protein at a ratio of 10%(*v*/*v*). Crystals were grown in 0.1 *M* HEPES pH 7, 14% PEG 8000, 0.1 *M* MgCl_2_ in 1–1.5 µl hanging drops using the vapour-diffusion method. The crystals were transferred to a cryoprotectant solution consisting of the mother liquor supplemented with 20%(*v*/*v*) glycerol (S. Aumonier, personal communication).

### 2.2. Data collection   

#### 2.2.1. Thermolysin and urease   

The thermolysin data described in Zander *et al.* (2016 ▸) (Table 1 ▸) were re-analyzed and were reprocessed using *XDS* v.20180126. These data consisted of four different *MeshAndCollect* workflows on four different samples of thermolysin (Zander *et al.*, 2015 ▸). The urease sub-data sets from Zander *et al.* (2016 ▸) were re-analyzed for anomalous phasing without modification.

#### 2.2.2. Cerulean   

Cerulean data were collected at 100 K using a Dectris PILATUS3 6M detector on the ID30B MAD beamline at 6.0 keV at the European Synchrotron Radiation Facility (Mueller-Dieckmann *et al.*, 2015 ▸) (Table 1 ▸). Data collection was performed using the *MeshAndCollect* workflow (Zander *et al.*, 2015 ▸). This resulted in 480 sub-data sets in four mesh scans that were processed by *XDS* (Kabsch, 2010 ▸).

### 2.3. Genetic algorithm   

Our GA implementation is based on *DEAP* (https://github.com/DEAP/deap) as described previously (Zander *et al.*, 2016 ▸). However, the Python script was modified to use the overall statistics rather than the inner-shell statistics for all metrics except the *R*_meas_ value. The genetic algorithm uses default probabilities for mutation and crossover: 0.6 and 0.3, respectively. The overall target function is 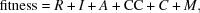
where 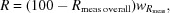


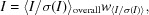


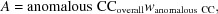


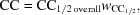


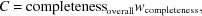


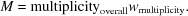
The weighting terms 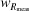, *w*_〈*I*/σ(*I*)〉_, *w*_anomalous CC_, 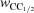, *w*_completeness_ and *w*_multiplicity_ can be any floating-point value and are by default 1.0, 2.0, 0.0, 1.0, 0.2 and 0.0, respectively. In these tests, a simple weight-balancing scheme was employed based on the statistical values after a single optimization cycle. Specifically, an *R*_user_ value is specified by the user to which the other components are scaled. The optimization is then run for a single cycle. The best values from each term are then obtained. The weighting scores are then computed as *w*_〈*I*/σ(*I*)〉_ = *R*_user_/〈*I*/σ(*I*)〉_overall best_, *w*_anomalous CC_ = *R*_user_/anomalous CC_overall best_, 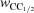 = *R*_user_/CC_1/2 overall best_ and *w*_completeness_ = *R*_user_/completeness_overall best_. While this method has the major drawbacks of not being based on the fully optimized values and requiring some knowledge of *R*_meas_, it nevertheless mitigates the domination of the fitness function by a single term. Automatic balancing of the weights brings the terms to approximately equal weights. Since in many cases, and in particular in this study, we wish to emphasize the importance of one term (*i.e.* the anomalous signal), user-specified weights than can be applied by multiplying the user weight by the automatic weight.

Normally, intermediate generations in GA optimization are deleted. However, we realized that the large number of combinations of sub-data sets calculated during the course of the GA optimization could provide more data with which to explore the relationships between merging statistics and downstream metrics (such as anomalous difference map peak heights, substructure correctness and weighted mean phase errors against refined structures). We therefore disabled the deletion of intermediate solutions. As a control, this was also performed with the GA mutation and crossover functions set to a probability of 0, and the selection scheme changed from fitness-based to random.

The preparation of *F*_A_ values was performed using *SHELXC* (Sheldrick, 2010 ▸). Reference structures for anomalous difference map and phase-error calculations were created by downloading PDB files 2wso for Cerulean (Lelimousin *et al.*, 2009 ▸), 4ceu for urease (Benini *et al.*, 2014 ▸) and 3zi6 for thermolysin (Ferrer *et al.*, 2013 ▸), followed by several rounds of refinement in *REFMAC*5 (Murshudov *et al.*, 2011 ▸) and manual rebuilding in *Coot*. A final refinement in *PDB-REDO* was then performed (Joosten *et al.*, 2014 ▸). The refined reference structures for thermolysin (*R*_cryst_ = 16.8%, *R*_free_ = 19.7%), Cerulean (*R*_cryst_ = 17.4%, *R*_free_ = 22.5%) and urease (*R*_cryst_ = 16.5%, *R*_free_ = 19.8%) were used to calculate model-phased anomalous difference maps using *ANODE* (Thorn & Sheldrick, 2011 ▸).

### 2.4. Substructure determination and phasing   

*F*_A_ values and amplitudes from *SHELXC* were then used in *SHELXD* and *SHELXE* for phasing (Sheldrick, 2010 ▸). The *SHELXD* settings for thermolysin were FIND 5, NTRY 8000, SHEL 50 2.5. For urease and Cerulean, different strategies were tried with different resolution cutoffs, atom numbers and NTRY keywords.

## 3. Results and discussion   

### 3.1. Thermolysin   

#### 3.1.1. Merging statistics and anomalous peak height   

**Figure 1:**
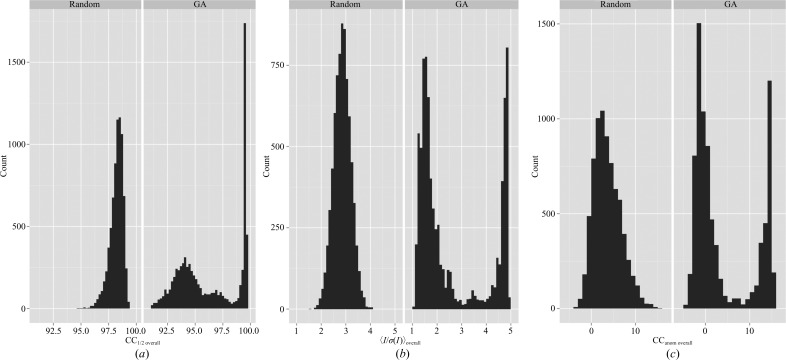
Improvement of CC_1/2 overall_, 〈*I*/σ(*I*)〉_overall_ and CC_anom overall_ by a GA for thermolysin. The distributions of 7500 merged data sets obtained by randomly selecting sub-data sets are shown on the left. GA results for 150 cycles × 50 individuals (7500 total evaluations) of GA optimization are shown on the right. For these data, improvement of the best merging groups occurred at the expense of the merging statistics of the worst merging groups. (*a*) CC_1/2 overall_, (*b*) 〈*I*/σ(*I*)〉_overall_, (*c*) CC_anom overall_.

**Figure 2:**
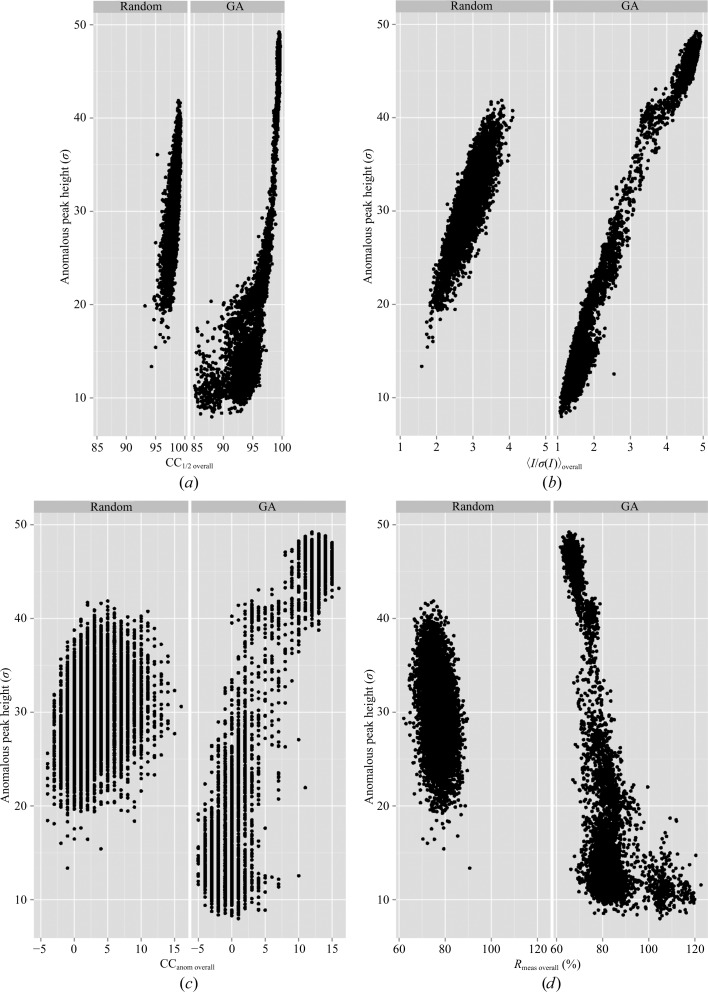
Improvement of anomalous signal by GA optimization of merging parameters for thermolysin. Anomalous peak heights in standard deviations (σ) above the mean value are shown on the *y* axis and merging statistic values are shown on the *x* axis. In each case, the left panel shows the results for randomly assembling merging groups. The right panel shows the results from GA optimization. The merging statistics are (*a*) CC_1/2 overall_, (*b*) 〈*I*/σ(*I*)〉_overall_, (*c*) CC_anom overall_ and (*d*) *R*_meas overall_ versus peak height.

Merging all data produced a data set (All_T) with an extremely poor *R*_meas overall_ value of 71.4% (Table 2 ▸). By contrast, 〈*I*/σ(*I*)〉_overall_ was 9.7, CC_anom overall_ was 20% and CC_1/2 overall_ was 99.3%. Randomly selecting data sets to create 7500 merged data sets produced 〈*I*/σ(*I*)〉_overall_ values between 1.6 and 4.2 (Fig. 1 ▸, left panel). In order to improve this signal further and to explore the relationship between anomalous peak height and various indicators of data quality, we optimized the grouping of these 158 sub-data sets using a GA. The algorithm was run for 150 cycles with a population of 50 individuals. This produced an improvement in the maximal 〈*I*/σ(*I*)〉_overall_ and CC_1/2 overall_ (Fig. 1 ▸), as well as other merging statistics, showing the efficacy of the GA method in improving these merging statistics. The best merging group (GA_T) showed improvements in the CC_1/2 overall_ (99.6%), CC_anom overall_ (22%) and *R*_meas overall_ (58.8%) values, but 〈*I*/σ(*I*)〉_overall_ was unchanged (9.7) compared with merging all sub-data sets. In order to explore the relationship between merging statistics and anomalous signal, we next looked at the relationship between model-phased anomalous peak heights and merging statistics. For this analysis, all intermediate GA solutions were used as input for *SHELXC* and *ANODE*. The individual merging statistics were then plotted as a function of anomalous peak height (Fig. 2 ▸). Nearly all merging statistics provided a good correlation with anomalous peak height. However, the *R*_meas_ values would normally all be deemed to be unacceptably high. Despite this fact, the mean anomalous peak height for *R*_meas overall_ values in the range 50–70% was 46 standard deviations above the mean density value (σ). Similarly, CC_1/2 overall_ has a strong correlation with anomalous peak height, but only at higher values (above 95%).

#### 3.1.2. Substructure determination and phasing   

**Figure 3:**
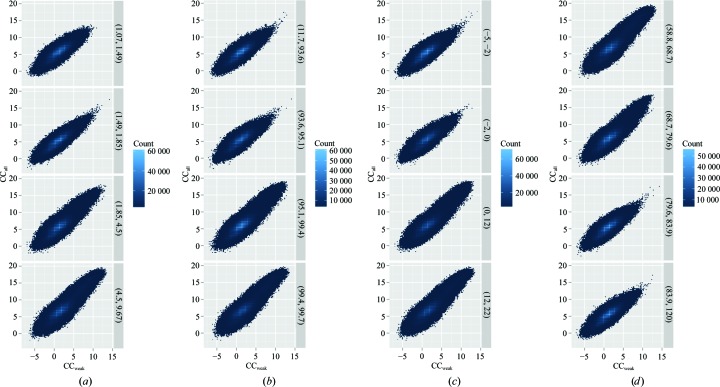
*SHELXD* substructure determination in thermolysin is more successful in data sets with higher (*a*) 〈*I*/σ(*I*)〉_overall_, (*b*) CC_1/2 overall_ and (*c*) CC_anom overall_ and (*d*) lower *R*_meas overall_. Because of the large number of data points (23 million), overlapping CC_all_/CC_weak_ values are colour coded according to the density of points. Solutions are divided according to their merging values. The ranges are shown on the right-hand side of the plots.

**Figure 4:**
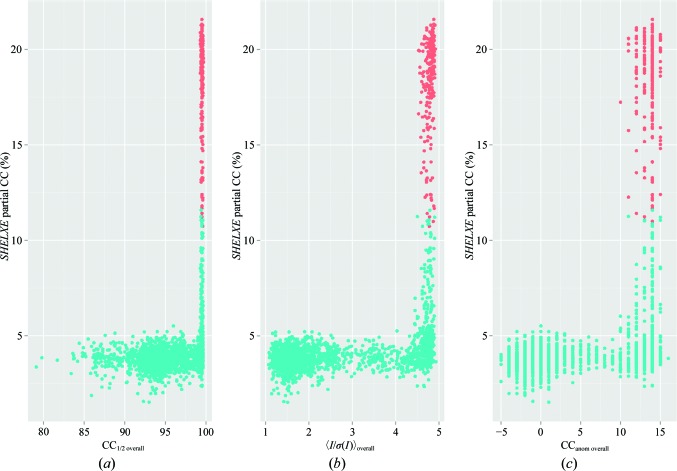
Merging statistics versus phasing outcome in thermolysin. Substructures from *SHELXD* were subjected to phasing and model building by *SHELXE*, and the CCs of automatically built main-chain atoms with the native data were evaluated as a function of the merging statistic. Furthermore, the best phase set from each *SHELXE* run was compared with a refined model to obtain phase errors. Data sets with weighted mean-phase error (wMPE) ≥ 40° are shown as cyan dots and those with wMPE < 40° are shown as orange dots. (*a*) CC_1/2 overall_, (*b*) 〈*I*/σ(*I*)〉_overall_, (*c*) CC_anom overall_.

**Figure 5:**
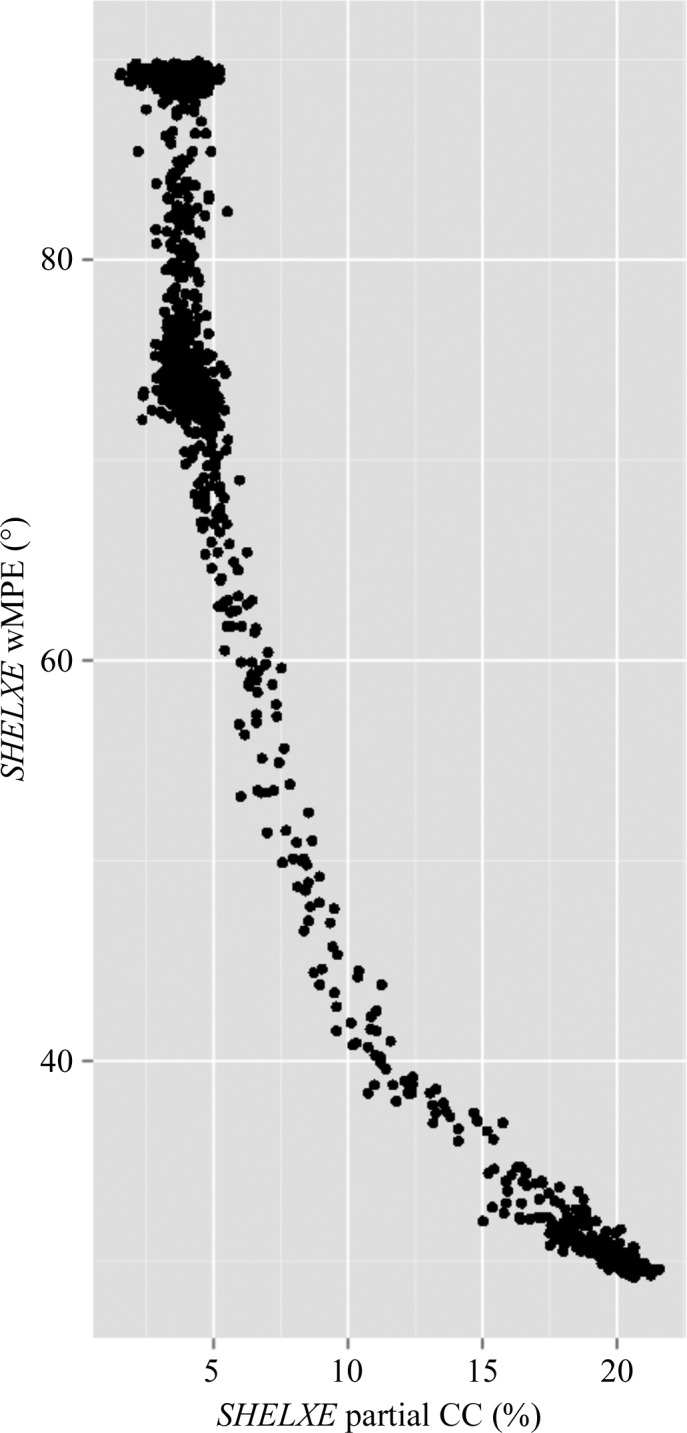
*SHELXE* CC of automatically built main-chain atoms using the native data versus weighted mean-phase error (wMPE) in thermolysin.

Despite large anomalous peak heights for many merged data sets (even merging all data produced a maximal anomalous difference map peak height of 46σ), structure solution was not straightforward. For both the All_T data set and the best GA data (GA_T), the position of the Zn atom could easily be determined. The four calcium sites had significantly lower peak heights and could only be found in anomalous residual maps. *SHELXD* was run for ∼5500 of the intermediate GA solutions and all of the resultant substructure-solution statistics were evaluated. The success of substructure determination, as evaluated by a plot of CC_all_ versus CC_weak_ from *SHELXD*, showed a clear trend of larger CC_all_/CC_weak_ values for data sets with higher 〈*I*/σ(*I*)〉_overall_, CC_anom overall_ and CC_1/2 overall_ values and lower *R*_meas overall_ values (Fig. 3 ▸). Phasing by *SHELXE* was possible for both the All_T data set and the GA_T data, but the GA data required only four rounds of solvent flattening/automatic building to obtain a partial CC value of >25%, while the ‘all data’ data set required eight rounds. High-quality models with partial CC values of 34% were produced from both data sets. In order to further examine the relationship between merging statistics and phasing, we ran *SHELXE* for all of the data sets for which *SHELXD* had been run. This extremely large scale set of phasing data was analysed in order to determine which merging statistics are correlated with phasing success. As in previous steps, we observed that CC_1/2 overall_, 〈*I*/σ(*I*)〉_overall_ and anomalous CC all correlated well with the successful phasing of structures (Fig. 4 ▸). Previous studies and anecdotal evidence have suggested that one of the most reliable metrics of phasing in *SHELXE* is the correlation coefficient of the partially automatically built model with the native data (Usón & Sheldrick, 2018 ▸). A threshold of 25% has been given as a cutoff value above which the structure is likely to be solved. We therefore plotted this metric against the weighted mean-phase error (wMPE) and were surprised to see that excellent wMPE values could be obtained even at CC values of 12% (Fig. 5 ▸). This reinforces the rule that at a CC of 25% the structure is almost certainly solved, but also suggests that values as low as 10% are worth examining in more detail.

### 3.2. Cerulean   

The fluorescent protein Cerulean is a 239-residue protein from the green fluorescent protein family with five S atoms. The very small number of S atoms compared with the number of amino-acid residues makes *de novo* phasing of this protein extremely difficult, but it represents a good test case for the improvement of weak anomalous signals.

#### 3.2.1. Merging statistics and anomalous peak height   

**Figure 6:**
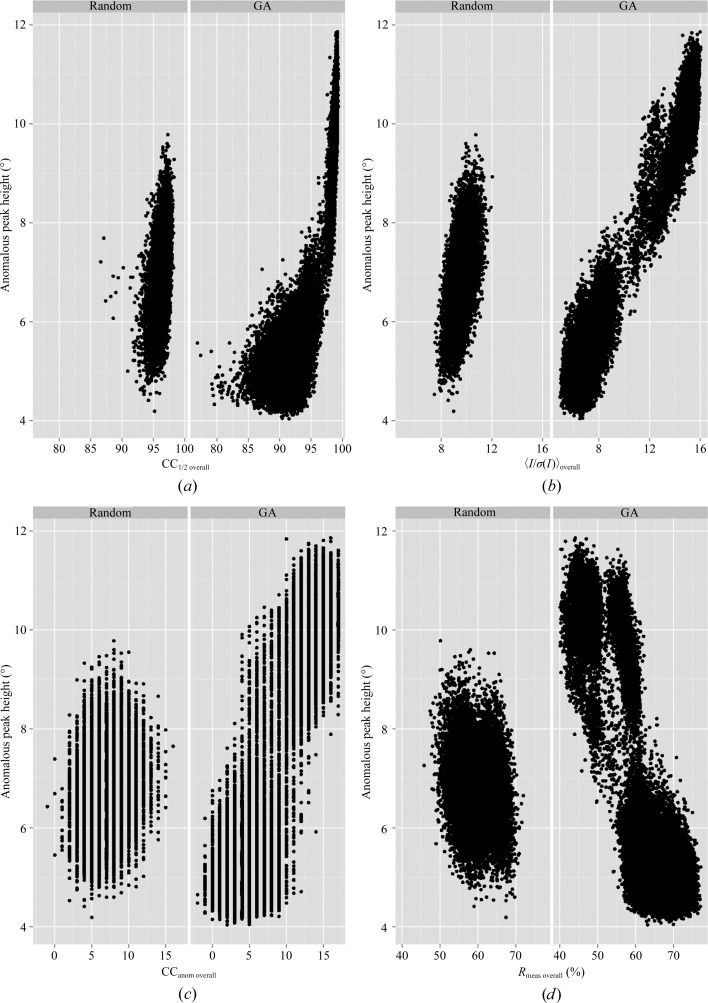
Improvement of anomalous signal by GA optimization of merging parameters for Cerulean. Anomalous peak heights in standard deviations (σ) above the mean value are shown on the *y* axis and merging statistic values are shown on the *x* axis. In each case, the left panel shows the results for randomly assembled merging groups. The right panel shows the results from GA optimization. The merging statistics are (*a*) CC_1/2 overall_, (*b*) 〈*I*/σ(*I*)〉_overall_, (*c*) CC_anom overall_ and (*d*) *R*_meas overall_ versus peak height.

Merging all data produced a data set (All_C) with an extremely poor *R*_meas overall_ value of 60.5% (Table 2 ▸). By contrast, 〈*I*/σ(*I*)〉_overall_ was 16.9, CC_anom overall_ was 9% and CC_1/2 overall_ was 98.6%. The GA was run for 500 cycles with a population of 25 individuals. This produced improvements in CC_1/2 overall_ (99.1%), *R*_meas overall_ (56.2%) and CC_anom overall_ (15%), but did not improve 〈*I*/σ(*I*)〉_overall_ (15.9). Despite these relatively modest gains in the merging statistics, the GA optimization yielded a significantly improved maximal anomalous peak height (11.8σ) compared with 9.7σ on merging all data (over S^γ^ of Cys170). The average anomalous peak heights were improved to 9.8σ for the cysteine S^γ^ atom and 9.2σ for the methionine S^δ^ atom, compared with 8.3σ and 8.2σ, respectively, when merging all data. As for thermolysin, all *XSCALE* merging runs were examined to explore the relationship between the merging statistics and anomalous peak heights (Fig. 6 ▸). In this case, the overall 〈*I*/σ(*I*)〉, CC_anom overall_ and CC_1/2 overall_ are also good choices for optimization. However, taken together with the fact that the 〈*I*/σ(*I*)〉_overall_ for merging all data was higher than that for the GA selected grouping (GA_C) but produced lower peak heights, it would be advisable to weight the CC_anom overall_ term highest.

#### 3.2.2. Substructure determination and phasing   

For both GA_C and All_C, we attempted to determine the substructure and calculate phases. To improve the chance of success, our attempts used two different programs: *SHELXD* and *HySS* from *PHENIX* (Grosse-Kunstleve & Adams, 2003 ▸; McCoy *et al.*, 2004 ▸). Although the peak heights obtained by GA optimization were reasonably high, the substructure could not be determined *de novo*, even when trying multiple low- and high-resolutions cutoffs (2.7–4.3 Å in 0.2 Å increments) and different numbers of atoms (five or seven) in *SHELXD* with NTRY = 30 000 for each attempt. We next attempted to identify a consensus solution within all of the *SHELXD* results using *SITCOM* (Dall’Antonia & Schneider, 2006 ▸). In post-analysis using *phenix.emma* to compare this consensus site with the known sites extracted from the refined structure, we retrieved a subset of four correct sites using the GA_C data set *versus* three using the All_C data set (Adams *et al.*, 2010 ▸; Grosse-Kunstleve & Adams, 2003 ▸). However, these four sites were distributed in a large list (82 total) of sites which was obtained by merging multiple coordinate files output from *SITCOM*. Therefore, identifying these correct sites without previous knowledge is unlikely. The most promising result was obtained for one *SHELXD* run, which resulted in two correct sites that were found using the GA_C data set. Despite this, it was not possible to optimize the substructure nor to determine the phases from this partial solution. Indeed, for both data sets (GA_C and All_C), even starting with the known substructure, obtaining interpretable phases using *SHELXE* or *Phaser* (0.4% solvent content and 2.2 and 2.19 Å resolution, respectively) was impossible (Read & McCoy, 2011 ▸). One possible explanation for this is that 〈*I*/σ(*I*)〉_overall_ was lower than for previously determined S-SAD proteins in general, including the previously described thermolysin data (Cianci *et al.*, 2008 ▸; Akey *et al.*, 2016 ▸).

### 3.3. Urease   

The *S. pasteurii* urease data described in Zander *et al.* (2016 ▸) were re-analyzed here, with a focus on anomalous phasing (Zander *et al.*, 2016 ▸). Urease has 29 S atoms in 26 methionines and three cysteines on three different polypeptide chains, with 790 amino acids in total.

#### 3.3.1. Merging statistics and anomalous peak height   

**Figure 7:**
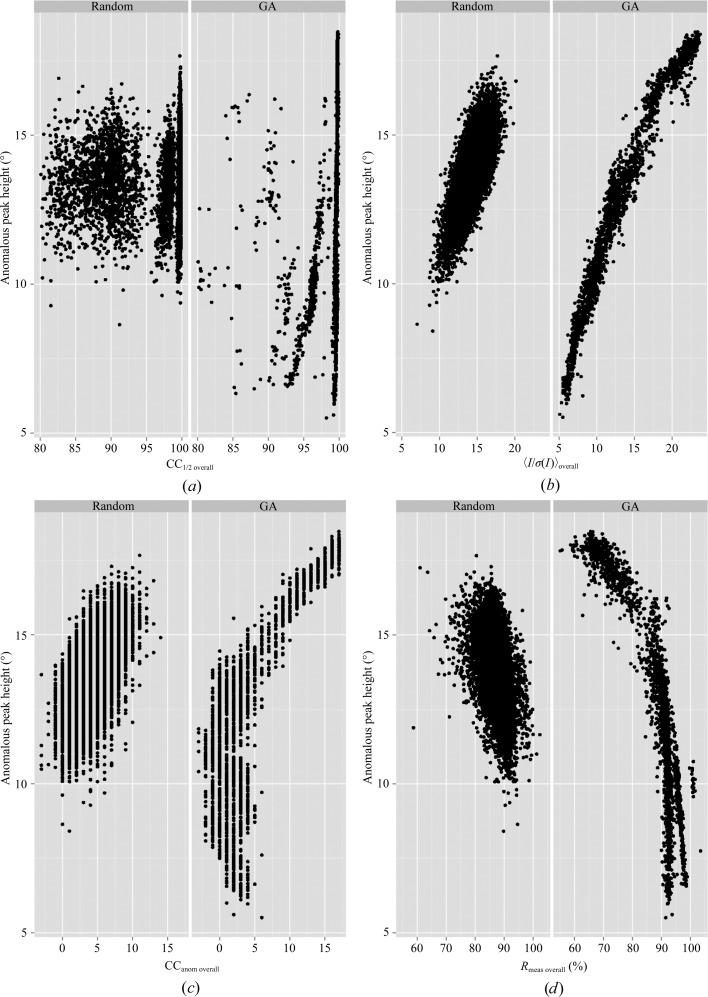
Improvement of anomalous signal by GA optimization of merging parameters for urease. Anomalous peak heights in standard deviations (σ) above the mean value are shown on the *y* axis and merging statistic values are shown on the *x* axis. In each case, the left panel shows the results for randomly assembled merging groups. The right panel shows the results from GA optimization. The merging statistics are (*a*) CC_1/2 overall_, (*b*) 〈*I*/σ(*I*)〉_overall_, (*c*) CC_anom overall_ and (*d*) *R*_meas overall_ versus peak height.

Merging all data (All_U) produced an *R*_meas overall_ value of 87.7%, an 〈*I*/σ(*I*)〉_overall_ of 24.6, a CC_1/2 overall_ of 99.9% and a CC_anom overall_ of 14% (Table 2 ▸). The GA was run for 150 cycles with a population of 30 individuals and three groups. This produced a data set with the same CC_1/2 overall_ (99.9%), a slightly worse 〈*I*/σ(*I*)〉_overall_ (23.7) and improvements to both *R*_meas overall_ (64.9%) and CC_anom overall_ (18%). Despite the similar statistics for CC_1/2 overall_ and 〈*I*/σ(*I*)〉_overall_, inspection of model-phased anomalous difference maps revealed increased peak heights for the GA data set (GA_U). While the All_U data set produced a maximal peak height of 16.6σ over Met479 and average values of 11.2σ and 7.7σ over methionine and cysteine residues, the GA_U data set produced a maximal peak height of 18.8σ over Met479 and 11.9σ and average values of 8.6σ over methionine and cysteine residues. As in the previous systems, we then examined the overall trends of merging statistics versus anomalous difference map peak heights (Fig. 7 ▸). As in Cerulean and thermolysin, a generally good correlation exists between all of the metrics and anomalous difference peak heights. However, CC_anom overall_ appears to be particularly useful in discriminating between the groupings that yield the highest anomalous difference peak heights.

#### 3.3.2. Substructure determination and phasing   

**Figure 8:**
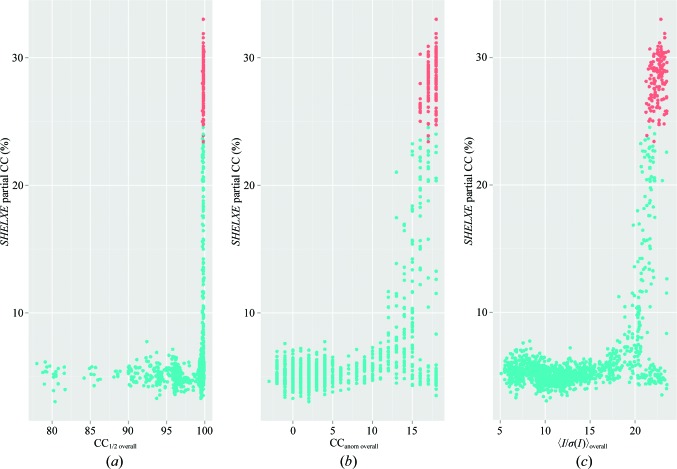
Merging statistics versus phasing outcome in urease. Known sulfur substructures were subjected to phasing and model building by *SHELXE*, and the CC of automatically built main-chain atoms with the native data was evaluated as a function of the merging statistic. The best phase set from each *SHELXE* run was compared with a refined model to obtain phase errors. Data sets with weighted mean-phase error (wMPE) ≥ 40° are shown as cyan dots and those with wMPE < 40° are shown as orange dots. (*a*) CC_1/2 overall_, (*b*) 〈*I*/σ(*I*)〉_overall_, (*c*) CC_anom overall_.

Despite the large anomalous difference map peak heights, the sulfur substructure could not be determined *de novo* using *SHELXD*, *PHENIX* or *PRASA* (Skubák, 2018 ▸). We used the same approach as that described for the Cerulean case but with NTRY = 50 000 and a set of high-resolution cutoffs from 2.5 to 3.9 Å every 0.2 Å. These produced multiple sets of possible substructures, which were then combined in *SITCOM* to identify the most represented sites. Unfortunately, very few sites from the known substructure could be found in the consensus substructure. The most promising individual *SHELXD* run resulted in five correct sites that were found using the GA_U data set, but this substructure could not be bootstrapped to successful phasing. Nevertheless, there was adequate signal in the data to determine high-quality phases starting from the known substructure (Fig. 8 ▸). Interestingly, when merging all data *SHELXE* could only produce a model with a partial CC of >20% (20.7%) after 14 macrocycles, whereas the GA selected data produced a model with a partial CC of 22% after only four macrocycles and of 29% after six macrocycles. This suggests that even improvements of a few percent in CC_anom overall_ can make a significant difference in phasing. A subset of the ∼12 000 intermediate merging runs (the total number is less than 30 × 150 × 3 because some individuals with the same grouping can occur during the GA evolution) were randomly selected for phasing starting from the known substructure. Specifically, 4300 merged groups were submitted to phasing and automatic building in *SHELXE*. Of these, 950 runs produced *SHELXE* partial CCs greater than 20%. Because CC_anom overall_ appears to be the most correlated to anomalous peak height at larger values (Section 3.3.1), we examined this merging metric and its relationship to how many *SHELXE* macrocycles of automatic building/solvent flattening were required for successful phasing. The minimum CC_anom overall_ that produced partial CCs of >20% was 13%, and this run required six iterations. The maximum CC_anom overall_ was 18%. There were 1856 data sets with a CC_anom overall_ of 18% and, of these, 57 could be solved with six iterations, 150 with five iterations, 389 with four iterations and 60 with three iterations. There was significant overlap between the most readily solved data sets (three, four and five iterations required) and the best GA merge, with an average similarity to the GA merge of 87%. It is not immediately obvious why the best overall *SHELXE* run required only three iterations compared with the best GA merge, which required four, despite both data sets having a CC_anom overall_ of 18% and having been started from the same substructure. Indeed, the best overall data set had a slightly lower maximal anomalous peak height (18.4) and slightly worse *R*_meas overall_ (66.2%), 〈*I*/σ(*I*)〉_overall_ (23.04) and CC_1/2 overall_ (99.7%) values compared with the GA. This suggests that for the purposes of optimization, a new metric for anomalous signal could be useful.

## 4. Conclusions   

Here, we have examined three experiments which combine relatively low anomalous signals with multi-crystal data collections. For thermolysin, urease and Cerulean, the estimated Bijvoet difference ratios are 1.7%, 1.3% and 1.1%, respectively. According to Olczak & Cianci (2018 ▸), phasing could be successful for the data sets presented here when 〈*I*/σ(*I*)〉_overall_ is in the range 23–69 for thermolysin, 30–90 for urease and 34–104 for Cerulean. It is therefore not surprising that *de novo* phasing was not possible for urease and Cerulean, given their overall 〈*I*/σ(*I*)〉_overall_ values of 24 and 16.9, respectively. However, it is somewhat surprising to see that a solution was found for thermolysin, albeit not in a straightforward manner, for which a data set with an 〈*I*/σ(*I*)〉_overall_ of only 9.7 could be assembled. Previously, thermolysin was solved by zinc SAD, with a similarly low estimated Bijvoet difference ratio of 1.1%, but with an 〈*I*/σ(*I*)〉_overall_ of 53.7 (Ferrer *et al.*, 2013 ▸). It is possible then that these estimates could be re-evaluated in the context of multi-crystal phasing.

One of the goals of this work was to study the connection between different merging statistics and multi-crystal phasing. Previous work by Terwilliger and coworkers and Zwart showed a strong correlation between CC_anom_ and experimental map correlation (Terwilliger *et al.*, 2016 ▸; Zwart, 2005 ▸). We used a similar approach, but took advantage of the fact that sub-data sets can be assembled into a large number of unique data sets. We could then analyse the relationship between the merging statistics for these data sets and the structure-solution statistics. This analysis showed that while most traditionally used merging statistics can be used in a GA target function to optimize anomalous signal, there are some particularities of multi-crystal data collections. In particular, *R* values that would normally indicate very poor quality data sets still produce large anomalous difference map peak heights and, in the case of thermolysin, *de novo* phases. This reinforces the notion that other merging indicators should generally be used in place of *R* values, and that this is especially true for multi-crystal data. Interestingly, in all three cases the GA data sets have a significantly reduced multiplicity (by a factor of 2) while retaining similar or better merging statistics. This suggests that non-isomorphism plays an appreciable role that is perhaps even larger than that observed in previous work (Assmann *et al.*, 2016 ▸). Indeed, it can be inferred that systematic errors play a significant role, because 〈*I*/σ(*I*)〉 should increase with greater multiplicity if the errors were predominantly random. A deeper analysis using recently developed methods for analysing the random and systematic error components of multi-crystal data will be pursued in future work (Diederichs, 2017 ▸). This will have implications on which metrics are the most suitable for inclusion in the GA target function, especially because the relationship between 〈*I*/σ(*I*)〉 and CC_1/2_ in particular can be quite different depending on the dominant type of error.

Nevertheless, for all three test cases CC_anom overall_ is correlated with the highest anomalous peak heights and is thus a logical target for GA or other optimization (Diederichs & Karplus, 2013 ▸). Indeed, differences of only a few percent in this value can make the difference between solving and not solving a structure. Increasing this signal, which is related to the strength of the anomalous signal and to the noise introduced by non-isomorphism, is not a new concept. However, the combination of a very large number of crystals with very weak anomalous signal is a relatively recent development. In this work, we have explored the connection between merging statistics and phasing for three such systems. In order to further improve multi-crystal phasing experiments and analysis, it will be necessary to revisit the origins of non-isomorphism and to perhaps identify new metrics for anomalous signal.

## Figures and Tables

**Table 1:** Data-collection parameters for mesh sub-data sets

	Thermolysin	Cerulean	Urease
Space group	*P*6_1_22	*P*2_1_2_1_2_1_	*P*6_3_22
Unit-cell parameters (Å)	*a* = 92.9, *b* = 92.9, *c* = 129.5	*a* = 53.1, *b* = 62.6, *c* = 69.8	*a* = *b* = 132.43, *c* = 190.6
Beamline	ID29, ESRF	ID30B, ESRF	P13, PETRA III
Wavelength (Å)	1.280	2.0664	2.0664
Beam diameter (horizontal × vertical or diameter) (µm)	10 × 10	20	30
Crystal size range (µm)	20 × 20 × 100	10 × 10 × 20	20 × 20 × 40–70
Photon flux (photons s^−1^)	4.1 × 10^11^, 8.4 × 10^11^	3.45 × 10^11^	3.4 × 10^11^
Exposure per image (s)	0.037	0.1	0.04
No. of images	100	100	300
Dose per sub-data set (average diffraction-weighted dose) (MGy)	3.0–6.2	9.02	0.48
Dose per sub-data set (average dose to exposed region) (MGy)	4.2–8.7	13.65	0.82
Oscillation range (°)	0.1	0.1	0.1
Total angular range per sub-data set (°)	10	10	30

**Table 2:** Data-collection merging statistics All_T, data set obtained using all of the sub-data sets collected from thermolysin micro-crystals; GA_T, data set obtained by the selection of sub-data sets by the GA. All_C, data set obtained using all of the sub-data sets collected from Cerulean micro-crystals; GA_C, data set obtained by the selection of sub-data sets by the GA. All_U, data set obtained using all of the sub-data sets collected from urease micro-crystals; GA_U, data set obtained with a selection of sub-data set by the GA.

	Thermolysin†		Cerulean		Urease†	
	All_T	GA_T	All_C	GA_C	All_U	GA_U
No. of sub-data sets	158	89	481	244	127	65
GA population size	—	50	—	25	—	30
GA generations	—	150	—	500	—	150
GA *R* weight	—	1 (default)	—	1 (default)	—	1 (default)
GA *I* weight	—	800	—	300	—	2000
GA CC_1/2_ weight	—	0 (default)	—	100	—	500
GA groups	—	3 (default)	—	3 (default)	—	3 (default)
Sub-data-set *R*_meas inner_ (average, standard deviation) (%)	29.8, 34.7	18.7, 22.8	43.7, 55.9	34.7, 52.6	15.3, 18.9	7.5, 9.5
Sub-data-set 〈*I*/σ(*I*)〉_inner_ (average, standard deviation)	7.4, 6.0	10.1, 6.0	5.0, 4.1	5.8, 4.5	10.9, 6.7	14.8, 6.0
Sub-data-set completeness (average, standard deviation) (%)	45.0, 4.6	45.4, 4.4	16.7, 2.1	16.9, 1.9	42.9, 4.9	42.8, 4.1
Resolution range (Å)	100–1.65	100–1.65	100–2.19	100–2.2	100–2.1	100–2.1
Total No. of reflections	6376244	3539280	1439894	744145	20422032	10464700
No. of unique reflections	75325	75344	22344	22167	109116	108537
Completeness (inner, outer, overall) (%)	99.6, 100, 100	99.5, 100, 100	100, 58.1, 96.4	100, 9.2, 91.0	99.9, 99.4, 100	99.9, 81.3, 98.6
Multiplicity (inner, outer, overall)	96.67, 81.85, 84.65	53.34, 45.59, 46.97	86.12, 9.4, 64.44	43.82, 3.102, 33.156	230.61, 326.48, 187.15	118.17, 38.01, 96.41
*R*_merge_ ‡ (inner, outer, overall) (%)	41.1, 426.5, 71.0	30.3, 462.6, 58.2	66.9, 88.2, 60.1	66.1, 66.6, 55.5	94.8, 159.1, 87.5	81.6, 135.1, 64.6
*R*_meas_ ‡ (inner, outer, overall) (%)	41.4, 429.1, 71.4	30.6, 467.7, 58.8	67.3, 92.6, 60.5	66.9, 75.4, 56.2	95.0, 160.2, 87.7	81.9, 137.0, 64.9
〈*I*/σ(*I*)〉 (inner, outer, overall)	25.82, 1.4, 9.68	24.83, 1.57, 9.67	28.65, 2.71, 16.95	26.71, 1.98, 15.93	102.11, 2.69, 24.62	96.13, 2.55, 23.71
SigAno (inner, outer, overall)	1.443, 0.610, 0.921	1.402, 0.625, 0.932	1.755, 0.67, 0.971	1.651, 0.749, 0.938	3.435, 0.746, 1.077	3.518, 0.71, 1.016
CC_1/2_ (inner, outer, overall)	98.2, 61.2, 99.3	99.4, 61.8, 99.6	98.5, 60.8, 98.6	98.9, 50.3, 99.1	100, 64.2, 99.9	99.8, 58.1, 99.9
CC_anom_ (inner, outer, overall)	58, 4, 20	66, 1, 22	22, −10, 9	16, −17, 15	87.2, 0, 14	91, 3, 18

† From Zander *et al.* (2016 ▸).

‡ *R*_merge_ = 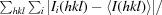 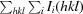 and *R*_meas_ = 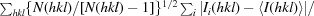 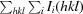.
